# Supplementary material for: Fostering proactive work behavior: Where to start?
Source: PLoS One. 2024 May 2;19(5):e0298936. doi: 10.1371/journal.pone.0298936 (PMC11065301; doi:10.1371/journal.pone.0298936)
Supplement: Attachment — Submitted filename: RESPONSES TO REVIEWERS.docx [file pone.0298936.s001.docx]

Reviewer #1:

The manuscript is interesting as they examined rehabilitation officers at the Narcotics Penitentiary in Sumatra, they presented very interesting fact, however the authors did not highlight the theoretical gap as the basis of developing their model.

Please add basic theories as the reference for the research design.

Methodology: please justify further on the samplng technique used. This sentence " proportionate purposive sampling approach that involved obtaining samples based on specific criteria" needs further clarification.

Lack of conistency on the terms used, which one is correct among these three: proactive behavior, proactive personality and proactive attitude, as those terms are different.

Dear reviewer,

Thank you for your comment.

Basic theories have been added in the Literature Review section.

The term *proportionate purposive sampling approach* has been revised.

The use of terms has been standardized.
