# Supplementary material for: Fostering proactive work behavior: Where to start?
Source: PLoS One. 2024 May 2;19(5):e0298936. doi: 10.1371/journal.pone.0298936 (PMC11065301; doi:10.1371/journal.pone.0298936)
Supplement: Attachment — Submitted filename: RESPONSES TO REVIEWER R2.docx [file pone.0298936.s002.docx]

**Reviewer #2:**

The abstract is important to represent the content of the article and it need to be improved. For example, the first part of the abstract should be improved providing a better introduction of the topic and the reason that you conduct this work.

Dear Reviewer,

Thank you for your comments.

Abstract has been improved as suggested

In introduction section it is good to express the need for the study with the backlog of literature exists in the framework. What is the scientific contribution of your paper to the science? Can you please indicate scientific implications of your paper?

Literature review: Please review the previous studies. The literature review does not show a research gap.

Dear Reviewer,

Thank you for your comments.

More statements have been added in the Introduction section. The research gap is also stated in the Introduction section.

How were the survey results verified?

The empirical section in many places, is devoid of interpretation and critical analysis of the research results. Most often, it is a description of the table's contents.

The results section should be separated from the discussion.

Discussion section: It would be appropriate to specify in more detail how this research differs from the already published paper that deals with a similar topic. To increase the significance of the results, the discussion part should embrace the differences and similarities among your findings and those of other scholars.

Dear Reviewer,

Thank you for your comments.

The Results section is intended to present the results of the analysis and findings. An in-depth interpretation of the results is presented in the Discussion section.

Conclusions: The section Conclusion can be expanded to include the limitations of this work and an indication of future research directions. Further applications of this proposed method should be discussed in detail, it may also good to add some real life cases, which can express the benefit of proposed method, further, and it may catch more attention.

Dear Reviewer,

Thank you for your comments.

The Conclusion section has been improved as suggested. The authors also decided to separate the Implication section into Theoretical Implication and Managerial Implication.
